# Peripheral Vascular Compression in a Patient With Diffuse Tenosynovial Giant Cell Tumor of the Knee: A Case Report Focusing on the Role of the Ultrasound

**DOI:** 10.7759/cureus.64836

**Published:** 2024-07-18

**Authors:** Delange Augustin, Delange Hendrick Augustin, Jefferson Arnold Théodas, Almenord Pharol, Clifford Georges Patrick Khawly

**Affiliations:** 1 Radiology, Orthocare+, Port-au-Prince, HTI; 2 Radiology, Hôpital de l'Université d'Etat d'Haïti, Port-au-Prince, HTI; 3 Orthopaedics and Traumatology, Orthocare+, Port-au-Prince, HTI; 4 Orthopaedics and Traumatology, Hopital Universitaire la Paix, Port-au-Prince, HTI; 5 Orthopaedics and Traumatology, Hopital Bernard Mevs, Port-au-Prince, HTI; 6 Orthopaedics and Traumatology, Hopital Universitaire de Mirebalais, Port-au-Prince, HTI

**Keywords:** vascular compression, joint swelling, ultrasound guided biopsy, knee ultrasound, tenosynovial giant cell tumor

## Abstract

Tenosynovial giant cell tumors (TGCTs) are benign histo-fibrocystic tumors originating from the synovium of joints, bursae, or tendon sheaths. They are categorized into localized and diffuse types, each with distinct clinical presentations and management approaches. The diffuse form, which is rare, generally affects a single joint and is characterized by joint swelling, pain, functional limitation, and often hemarthrosis. While MRI is commonly used for diagnosis, we present a case highlighting ultrasound's significance in diagnosing and managing TGCTs, particularly for identifying vascular complications. A 59-year-old female with a 10-year history of recurrent swelling, pain, and functional limitation of the right knee was evaluated for persistent symptoms and unilateral right peripheral edema. Ultrasound revealed multiple hypoechoic, vascularized masses with both homogeneous and heterogeneous echostructures and a significant suprapatellar effusion. An ultrasound-guided biopsy confirmed the diagnosis of a recurrent diffuse TGCT complicated by vascular compression of the popliteal vein. The patient underwent mass resections, total synovectomy, and radiotherapy to reduce the risk of recurrence.

Ultrasound is cost-effective and highly beneficial for the diagnosis, treatment planning, and monitoring of diffuse TGCTs. Total synovectomy combined with radiotherapy or intra-articular yttrium-90 injection is the preferred treatment to prevent recurrence and complications.

## Introduction

Tenosynovial giant cell tumors (TGCTs) are benign histo-fibrocystic tumors originating from the synovium of joints, bursae, or tendon sheaths [1-4]. Although TGCTs display identical histological characteristics, they are classified into two types: localized and diffuse, each associated with distinct clinical presentations and management strategies [2,5]. The diffuse form, which is rare, typically affects a single joint, either intra-articular or extra-articular. It is characterized by joint swelling, pain, functional limitation, and often hemarthrosis [6,7]. MRI is commonly used for diagnosis. However, ultrasound also plays a crucial role in describing the lesion and dynamically assessing its relationship to adjacent structures. These tumors generally present as hypoechoic, vascularized masses with a homogeneous echostructure, although they can sometimes appear heterogeneous. They remain non-mobile during the flexion or extension of adjacent tendons [6,8].

We present a case of a female with a history of recurrent swelling, pain, and functional limitation of the right knee with unilateral right peripheral edema, highlighting the challenges in managing TGCTs and the importance of ultrasound in diagnostic procedures, especially in diagnosing vascular complications.

## Case presentation

**Figure 1:**
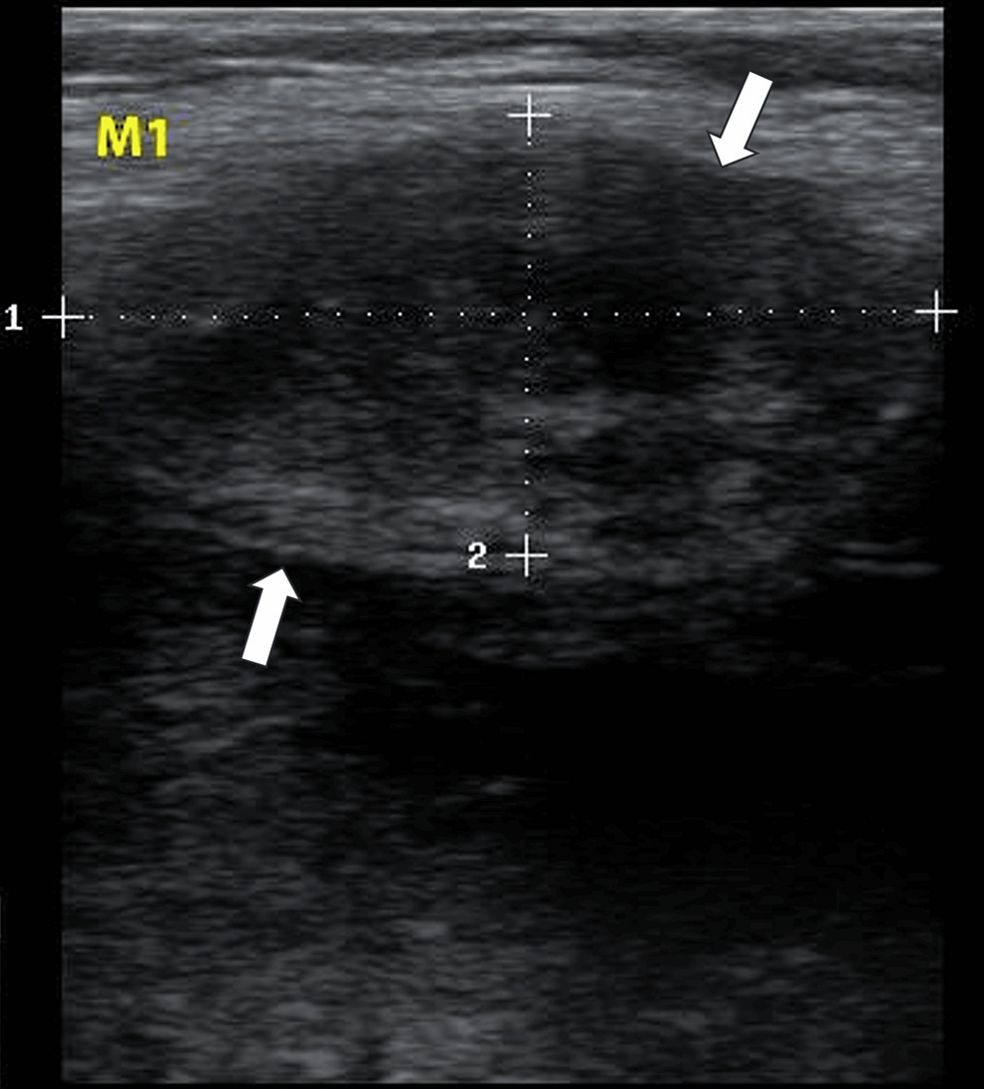
Ultrasound image of M1 The image shows a mass projecting from the tendinous sheath of the vastus lateralis, measuring 3.73 cm (dotted line 1) x 1.89 cm (dotted line 2)

**Figure 2:**
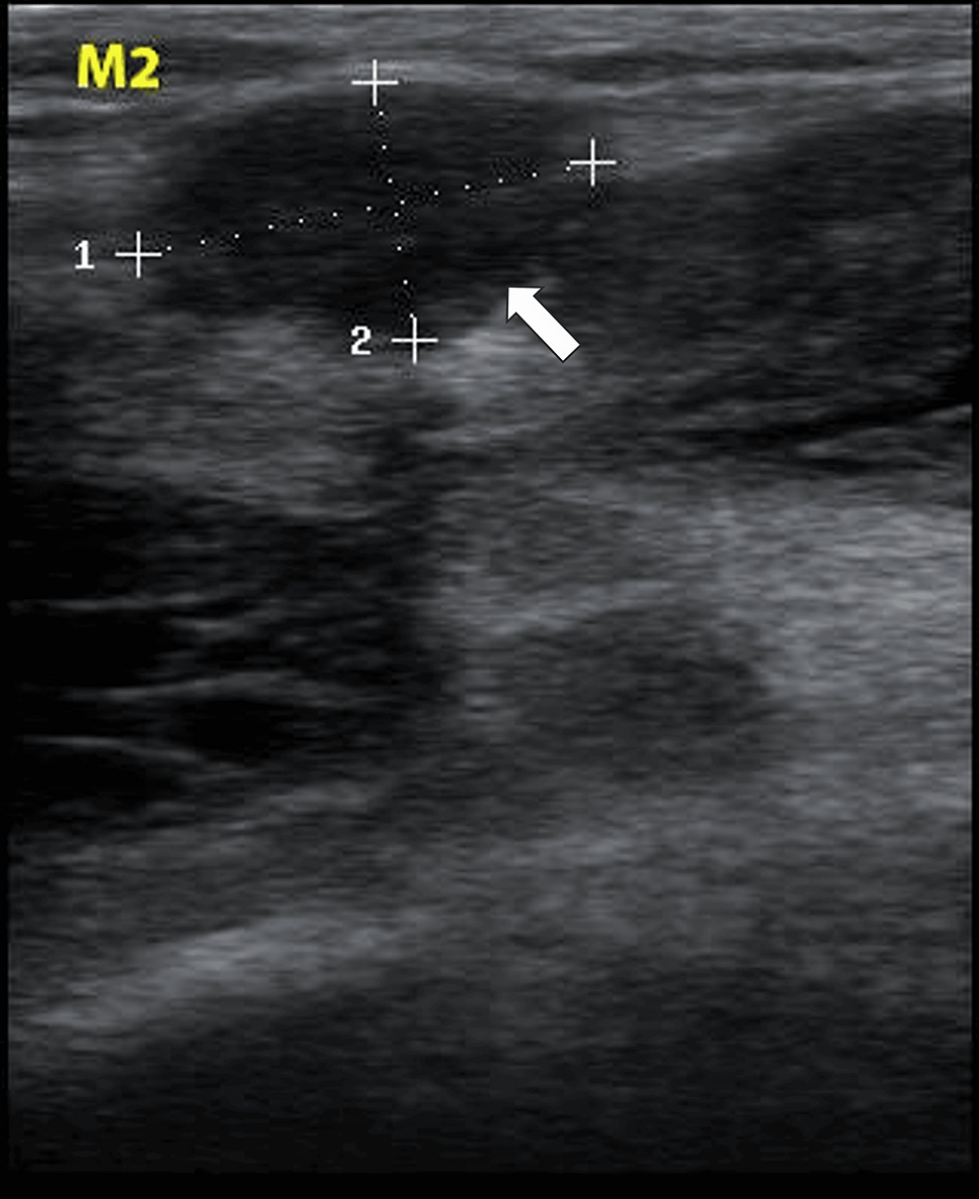
Ultrasound image of M2 The image shows a mass located in the suprapatellar compartment behind the tendon sheath of the vastus lateralis, measuring 1.81 cm (dotted line 1) x 1.02 cm (dotted line 2)

**Figure 3:**
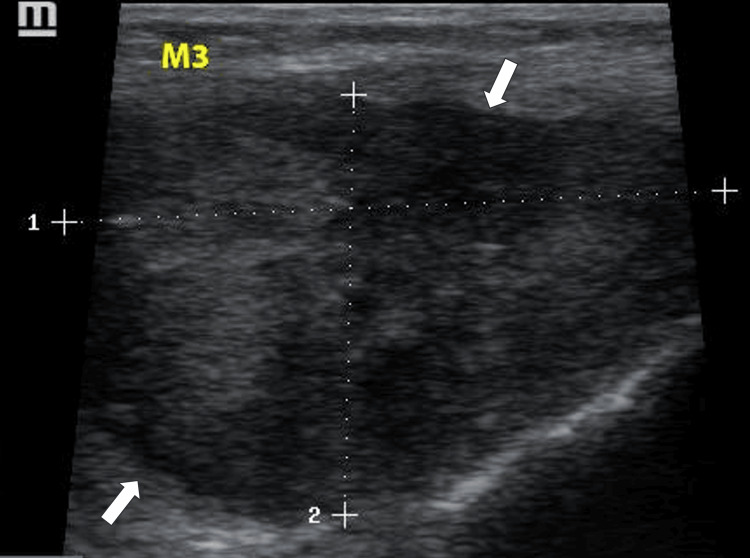
Ultrasound image of M3 The image shows a mass located in the suprapatellar compartment behind the tendon sheath of the vastus lateralis, measuring 4.56 cm (dotted line 1) x 2.80 cm (dotted line 2)

A 59-year-old woman, who had experienced recurrent swelling, pain, and functional limitation of the right knee for 10 years, was evaluated for persistent symptoms now accompanied by unilateral right peripheral edema. Five years earlier, she had undergone knee surgery where an unidentified mass was resected. A physical examination identified several firm masses protruding from the quadriceps and popliteal fossa and a positive pitting sign at the right ankle and leg. Ultrasound examination of the knee revealed multiple oval masses of varying sizes, hypoechoic and vascularized, with either homogeneous or heterogeneous echostructure and regular contours. These masses were non-mobile during knee flexion. One mass (M1), projecting from the tendinous sheath of the vastus lateralis, measured 3.73 x 1.89 cm (Figure 1). Two masses (M2, M3), located in the suprapatellar compartment behind the tendon sheath of the vastus lateralis, measured 1.81 x 1.02 cm and 4.56 x 2.80 cm, respectively (Figures 2, 3).

**Figure 4:**
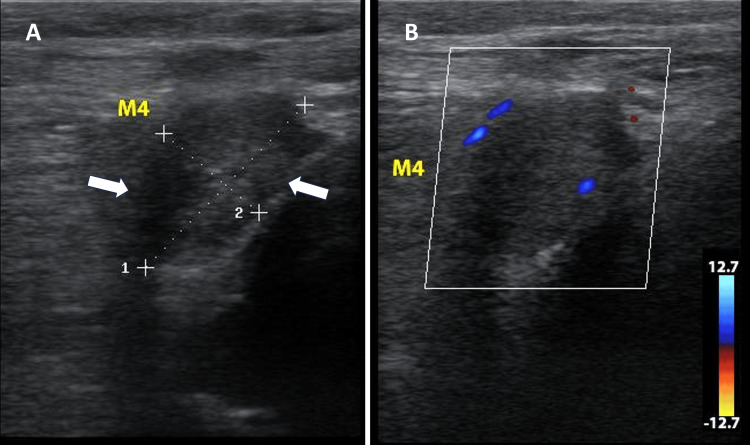
B-mode and color Doppler ultrasound images of M4 (A) B-mode ultrasound image of M4, a mass in the popliteal fossa, measuring 2.39 cm (dotted line 1) x 1.30 cm (dotted line 2). (B) Color Doppler ultrasound image of M4, showing vascularization within the mass in the popliteal fossa (blue and red coloration)

**Figure 5:**
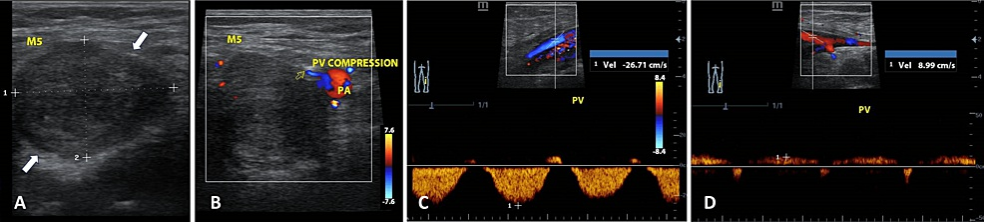
Various ultrasound images related to M5 (A) B-mode ultrasound image of M5, the largest mass in the popliteal fossa, measuring 3.39 cm (dotted line 1) x 2.51 cm (dotted line 2). (B) Color Doppler ultrasound image of M5, illustrating the relationship between the mass and the popliteal vein (PV) and popliteal artery (PA). (C) Ultrasound image showing the flow velocity of the popliteal vein (PV) at the compression zone caused by M5. (D) Ultrasound image of the popliteal vein (PV) flow velocity distal to M5, demonstrating decreased velocity

**Figure 6:**
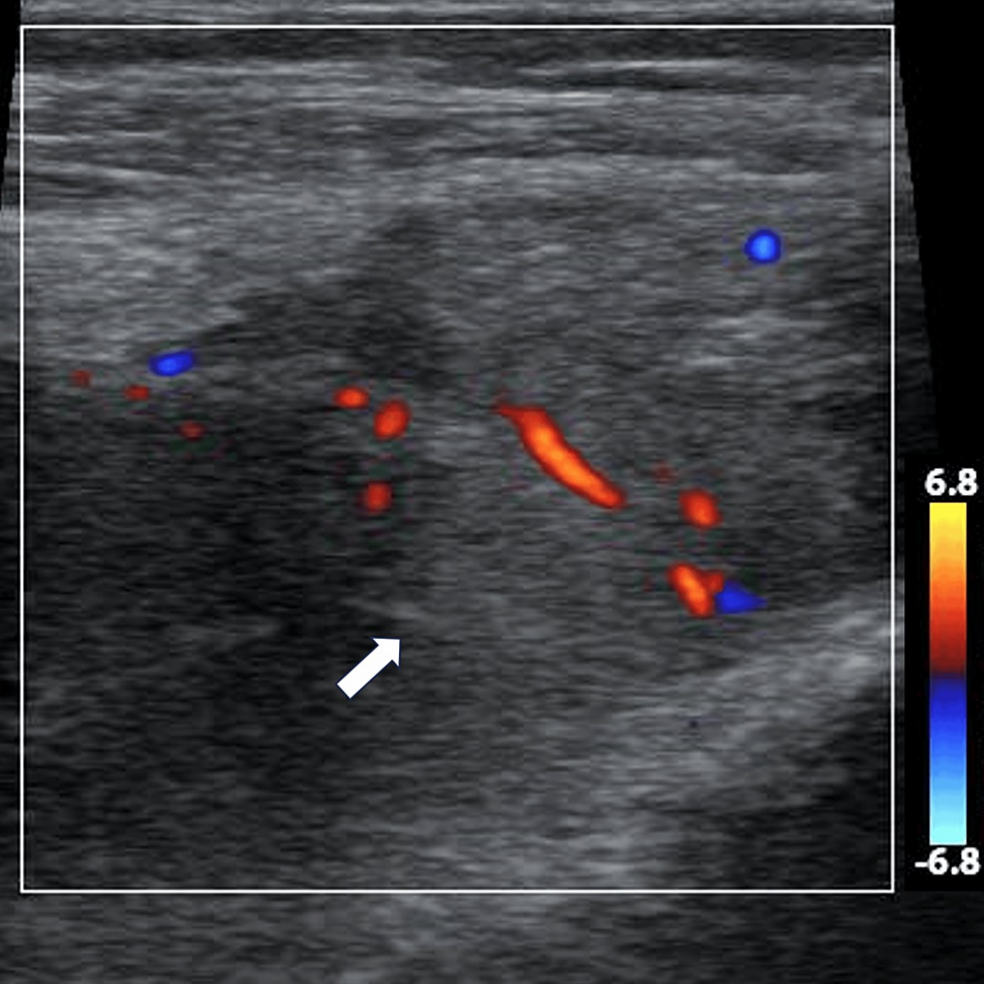
Ultrasound image of suprapatellar synovial hypertrophy with Color Doppler highlighting areas of hypervascularization

**Figure 7:**
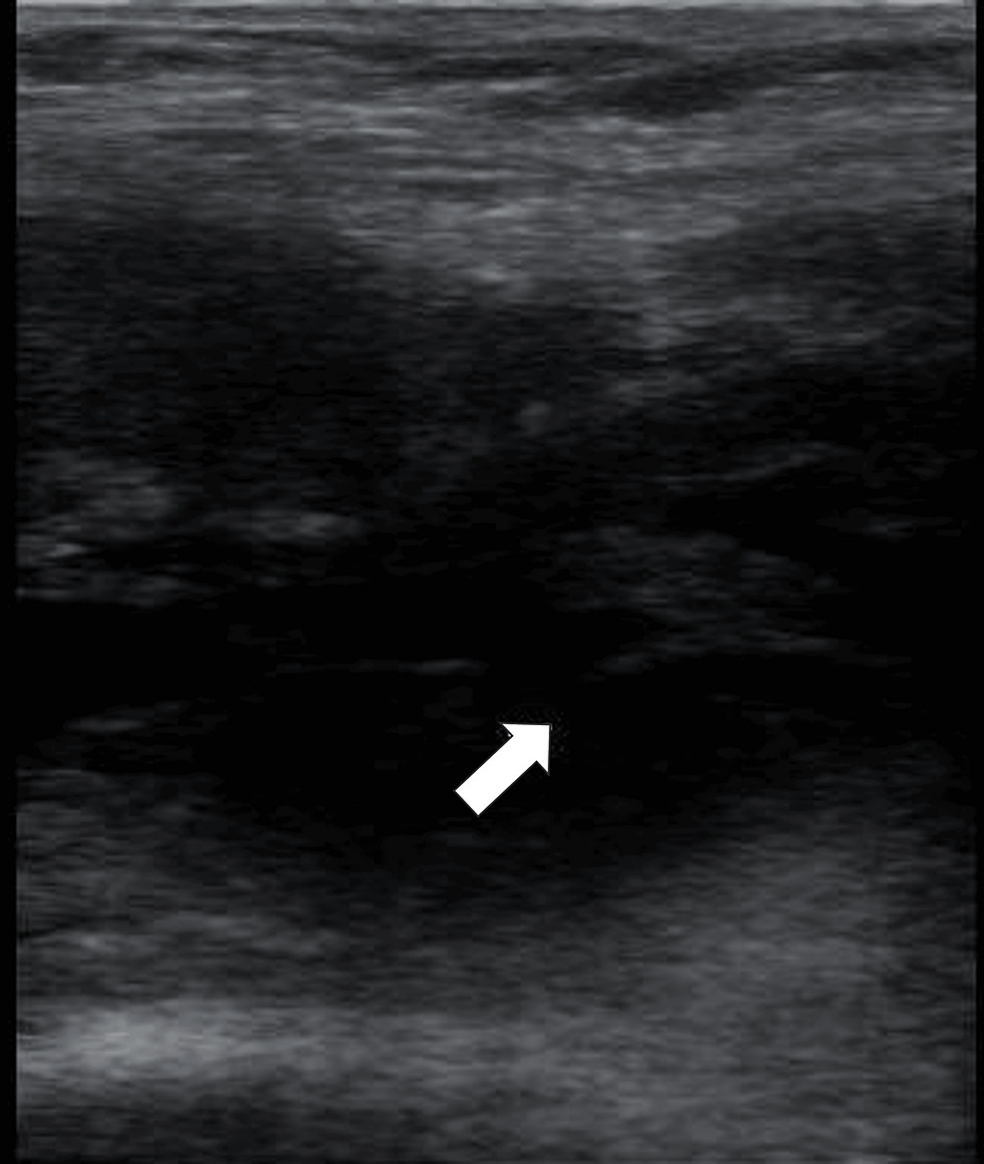
Ultrasound image of a suprapatellar hematoma, showing coagulation debris (arrow)

Two additional masses (M4, M5) were found in the popliteal fossa, with M4 measuring 2.39 x 1.30 cm (Figure 4) and the largest mass, M5, measuring 3.39 x 2.51 cm, compressing the popliteal vein. The venous blood flow velocity distal to M5 was 8.99 cm/s, increasing to 26.71 cm/s at the compression zone (Figure 5). A significant suprapatellar effusion was noted alongside areas of synovial hypertrophy and hypervascularization (Figure 6) and other areas with coagulation debris compatible with hematomas (Figure 7). Ultrasound-guided arthrocentesis evacuated more than 60 cc of thick, citrine-yellow synovial fluid. The ultrasound findings suggested a diagnosis of TGCT of the right knee with compression of the right popliteal vein. Differential diagnoses included multiple intramuscular myxomas and multiple fibroids of the tendon sheaths of the right knee.

An ultrasound-guided biopsy revealed histiocytic layers, pigmentary clusters, and multinucleated giant cells consistent with pigmented villonodular synovitis, confirming the ultrasound findings. Given the history of an unidentified mass resection at the knee, the diagnosis was revised to recurrent TGCT of the knee, complicated by vascular compression of the popliteal vein. The patient was referred for mass resections, total synovectomy, and radiotherapy to reduce the risk of recurrence.

## Discussion

TGCTs carry a high risk of recurrence [9]. There are documented cases of localized TGCTs undergoing aggressive and malignant transformations with potential for metastasis [3]. Timely and appropriate management is essential to prevent complications. Radiographic signs are generally nonspecific, primarily showing joint effusion. Bone density and joint space remain intact until the disease progresses to advanced stages, with no visible calcification. There may be extrinsic marginal erosions, and focal areas of soft tissue swelling around the joint may be dense due to hemosiderin deposition [7]. CT scans may reveal joint effusions. The enlarged synovial fluid can present as a soft tissue mass, appearing slightly hyperdense compared to adjacent muscle due to hemosiderin. Bone erosions are typically more apparent on CT. MRI often displays a mass-like synovial proliferation with lobulated or indistinct margins and low signal intensity on T1-weighted images, attributable to hemosiderin deposition [8].

There is scarce literature on the diagnostic use of ultrasound in these cases. Nonetheless, in our case, ultrasound findings were as pertinent, if not more so, as other radiological techniques. Significant ultrasound features included multiple hypoechoic, vascularized masses with either homogeneous or heterogeneous echostructures and regular contours projecting from the tendons and immobile during dynamic movements. Accompanying signs of joint effusion, hematomas, synovial hypertrophy, and hypervascularization corroborated the diagnosis.

These ultrasound findings substantially narrow down the differential diagnosis. One similar pathology is fibroma of the tendon sheath, which rarely presents with multiple masses and is typically confined to smaller joints without synovial lesions, unlike diffuse TGCTs that predominantly affect larger joints [2,10,11]. Another possible diagnosis is multiple intramuscular myxomas, characterized by hypoechoic or almost anechoic masses with heterogeneous echostructures, minimal or no vascularity, and well-defined contours localized in surrounding soft tissues. Many cases show posterior reinforcement, associated with the “sonographic bright ring sign” [12].

Given the recurrence rates ranging from 7% to 60%, averaging around 35%, ultrasound-guided biopsy is preferred over excisional biopsy to minimize surgical interventions and to direct the management toward complete synovectomy, which offers promising recovery outcomes. Total excision of the synovium can be challenging, which explains the frequent use of adjuvant therapies, especially external beam radiotherapy. Intra-articular injection of yttrium-90 is also a viable option [7,13].

## Conclusions

In managing diffuse TGCTs, ultrasound is highly valuable. It describes lesions and detects hypertrophy and hypervascularization of adjacent synovium, assesses the vascular status of surrounding vessels, and aids in guiding biopsy procedures. This method can be cost-effective and advantageous for diagnosis, treatment planning, and follow-up. Total synovectomy combined with radiotherapy or intra-articular yttrium-90 injection remains the preferred treatment to prevent recurrence and manage complications.
